# Regulatory Role of Host MicroRNAs in Flaviviruses Infection

**DOI:** 10.3389/fmicb.2022.869441

**Published:** 2022-04-11

**Authors:** Wenjun Cai, Yuhong Pan, Anchun Cheng, Mingshu Wang, Zhongqiong Yin, Renyong Jia

**Affiliations:** ^1^Research Center of Avian Disease, College of Veterinary Medicine, Sichuan Agricultural University, Chengdu, China; ^2^Institute of Preventive Veterinary Medicine, Sichuan Agricultural University, Chengdu, China; ^3^Key Laboratory of Animal Disease and Human Health of Sichuan Province, Chengdu, China

**Keywords:** flaviviruses, host miRNAs, host–virus interaction, virus replication, inflammatory

## Abstract

MicroRNAs (miRNAs) are small non-coding RNA that affect mRNA abundance or translation efficiency by binding to the 3′UTR of the mRNA of the target gene, thereby participating in multiple biological processes, including viral infection. *Flavivirus* genus consists of small, positive-stranded, single-stranded RNA viruses transmitted by arthropods, especially mosquitoes and ticks. The genus contains several globally significant human/animal pathogens, such as Dengue virus, Japanese encephalitis virus, West Nile virus, Zika virus, Yellow fever virus, Tick-borne encephalitis virus, and Tembusu virus. After flavivirus invades, the expression of host miRNA changes, exerting the immune escape mechanism to create an environment conducive to its survival, and the altered miRNA in turn affects the life cycle of the virus. Accumulated evidence suggests that host miRNAs influence flavivirus replication and host–virus interactions through direct binding of viral genomes or through virus-mediated host transcriptome changes. Furthermore, miRNA can also interweave with other non-coding RNAs, such as long non-coding RNA and circular RNA, to form an interaction network to regulate viral replication. A variety of non-coding RNAs produced by the virus itself exert similar function by interacting with cellular RNA and viral RNA. Understanding the interaction sites between non-coding RNA, especially miRNA, and virus/host genes will help us to find targets for antiviral drugs and viral therapy.

## Introduction

MicroRNAs (miRNA) is a class of small non-coding RNA, about 20–24 nucleotides in length. miRNA is first transcribed from the genome to form primary miRNA (pri-miRNA), and then cleaved by Drosha enzyme and Dicer enzyme successively to form mature miRNA. One strand of mature miRNA typically degrades mRNA or inhibits translation by binding to the 3′untranslated region (UTR) of the target gene (Bartel, 2004). Not only in diverse biological pathways, cellular miRNAs also play vital roles in virus-host interactions, which are post-transcriptional regulators in directing regulation of virus proliferation (Wong et al., 2020).

**FIGURE 1:**
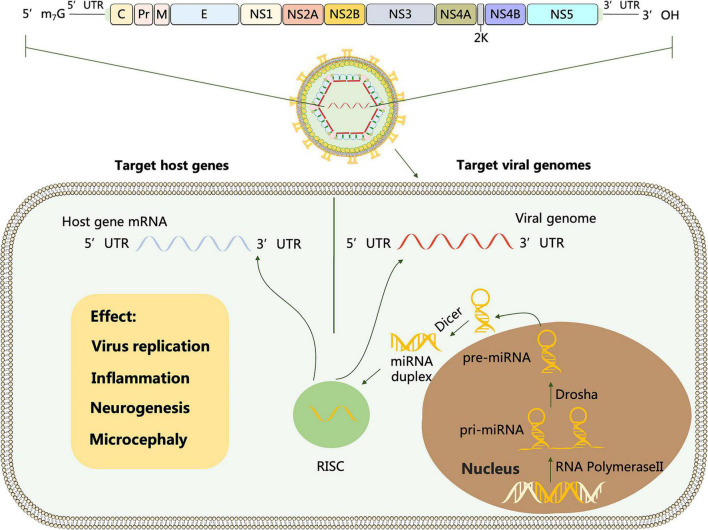
Schematic representation of flavivirus genome. The polyprotein encoded by the genome is cleaved by host and viral proteases to form three structural proteins: capsid (C), membrane (M) and envelope (E) and 7 non-structural proteins (NS-1, 2A, 2B, 3, 4A, 4B and 5). miRNA is transcribed from host mRNA to primary miRNA (pri-miRNA) in the nucleus by RNA polymerase II (Pol II). Pre-miRNA is transported from the nucleus to the cytoplasm and cleaved by the Dicer enzyme into miRNA double strands, one of which forms an RNA-induced silencing complex (RISC). Target viral genomes: After flavivirus infection, the RNA viral genome releases into the cytoplasm, and RISC carrying miRNA complements the non-coding and coding sequences of the virus through miRNA seed sequences to regulate viral replication. Target host genes: After flavivirus invasion, miRNAs can activate or repress specific cellular signaling pathways and ultimately mediate viral replication by regulating the target genes they interact with.

*Flavivirus* genus contains more than 50 arthropod-borne viruses (arboviruses), of which important family members include Dengue virus (DENV), Japanese encephalitis virus (JEV), West Nile virus (WNV), Zika virus (ZIKV), Yellow fever virus (YFV), Tick- borne encephalitis virus (TBEV) and Tambusu virus (TMUV). Flavivirus is a positive-sense RNA virus with a full length of about 11 kb. The structure of viral genome contains a single open reading frame (ORF) that encodes multiple proteins, cleaved by host and viral proteases into three structural proteins (C, prM, E) and seven non-structural proteins (NS1, NS2A, NS2B, NS3, NS4A, NS4B, and NS5), flanked by a 5′UTR and a 3′UTR, which is conserved throughout the *Flavivirus* genus (Heiss et al., 2011; Figure 1).

The expression of host miRNA can be altered by flavivirus infection (Campbell et al., 2014; Kumari et al., 2016; McLean et al., 2017; Cui et al., 2018; Bagasra et al., 2021). Further, differentially expressed miRNAs can directly regulate virus replication by complementing viral genome or indirectly by binding to crucial host genes, which has been reported in various viruses (Smith et al., 2012). For instance, Kumar et al. (2018) confirmed that miR-324-5p suppresses H5N1 highly pathogenic influenza A virus (HPAIV) replication through directly binding within the viral genome, thereby inhibiting viral gene expression. miR-140 inhibits classical swine fever virus (CSFV) genome amplification via interacting with an oncogene Rab25 in swine umbilical vein endothelial cells (Xu et al., 2020). Moreover, it is worth mentioning that miRNAs are usually negatively correlated with target genes. Nevertheless, miR-122 is a special case, as it specifically expresses and is highly abundant in human livers, while promoting the Hepatitis C virus (HCV) life cycle by targeting its 5′UTR (Jopling et al., 2005; Kunden et al., 2020).

Recently, interaction networks composed of non-coding RNAs, including miRNA, long non-coding RNA (lncRNA) and circular RNA (circRNA), have attracted increasing attention for their important roles in various biological processes such as viral infection. lncRNA/circRNA, as competing endogenous RNA (ceRNA), competitively bind to miRNA through their miRNA recognition element (MRE), thereby regulating the expression level of miRNA target mRNA (Salmena et al., 2011). Hence, lncRNA/circRNA–miRNA–mRNA interaction may be an important mechanism of flavivirus pathogenesis. In addition, the virus itself produces a variety of non-coding RNAs, such as subgenomic flaviviral RNAs (sfRNAs) and viral small RNAs (vsRNAs), the latter also include viral small interfering RNAs (siRNAs) and virus-encoded miRNAs. All of them can interact with cellular and viral RNA to regulate flavivirus replication (Schuessler et al., 2012; Hussain and Asgari, 2014; Zeng et al., 2021).

Based on the character that flavivirus completes its life cycle and pathogenicity is highly dependent on the metabolism of living host cells, clarifying the effect of host miRNA as well as other non-coding RNA on host–virus interactions will help us better understand the pathogenesis of flavivirus and novel candidates for the development of therapeutic interventions (Smith et al., 2017). In this review, we elaborate how miRNAs could relate to flavivirus infections and summarize the potential value of miRNAs in combating flavivirus.

## Regulation of miRNAs in Dengue Virus Infection

Dengue viruses are four closely related but antigenically distinct viral serotypes (DENV1, DENV2, DENV3, and DENV4) that cause very similar disease spectrum in humans. It is the tenth leading cause of death and morbidity in developing countries and the leading cause of death among children under 15 years of age in some South-East Asian countries (Lim, 2019). At present, DENV is also the most widely studied virus in the *Flavivirus* genus and has been reported to regulate its own replication by using host miRNA (Zhou et al., 2014; Castillo et al., 2016; Diosa-Toro et al., 2017; Kanokudom et al., 2017; Mishra et al., 2020).

### miRNAs Regulate Dengue Virus Proliferation via Directly Targeting Viral Genomes

Several independent studies have suggested that miRNAs can complementarily bind to non-coding regions or even coding regions of DENV genome and consequently influencing viral replication, while other research results imply that DENV infection regulates cellular miRNAs expression for their own interests (Miranda et al., 2006; Pasquinelli, 2012; Yan et al., 2014; Zhou et al., 2014; Wen et al., 2015; Castillo et al., 2016; Castrillón-Betancur and Urcuqui-Inchima, 2017; Kanokudom et al., 2017; Table 1).

**TABLE 1:** Differentially expressed miRNAs involved in DENV infection and their targets^a^.

MicroRNA	Virus-types	Host system	Target	Effect	References
↓ miR-484	DENV-1, DENV-2 (New Guinea C strain), DENV-4	Vero	↑ Viral 3′UTR	Promote virus replication	Castrillón-Betancur and Urcuqui-Inchima, 2017
↓ miR-744	DENV-1, DENV-2 (New Guinea C strain), DENV-4	Vero	↑ Viral 3′UTR	Promote virus replication	Castrillón-Betancur and Urcuqui-Inchima, 2017
↓ miR-133a	DENV-1, DENV-2 (New Guinea C strain), DENV-4	Vero	↑ Viral 3′UTR	Promote virus replication	Castillo et al., 2016
↑ miR-548 g-3p	DENV-1 (Hawaii strain), DENV-2 (New Guinea C strain), DENV-3 (Philippine H87 strain), DENV-4 (GZ/9809/2012 strain)	U937	↓ Viral 5’UTR	Inhibit virus replication	Wen et al., 2015
↑ aae-miR-281	DENV-2 (New Guinea C strain)	C6/36	↑ Viral 5′UTR	Promote virus replication	Zhou et al., 2014
↑ miR-21	DENV-2 (16681 strain)	HepG2	Viral NS1 (predictive)	Promote virus replication	Miranda et al., 2006; Kanokudom et al., 2017
↑ aae-miR-252	DENV-2 (New Guinea C strain)	C6/36	↓ Viral E	Inhibit virus replication	Yan et al., 2014
↓ miR-223	DENV-2 (TR1751 strain)	EAhy926	↑ STMN1	Promote virus replication	Wu et al., 2014
↑ miR-146a	DENV-2 (New Guinea C strain)	Primary human monocytes, THP-1	↓ TRAF6	Promote virus replication Biomarker	Wu et al., 2013; Xie et al., 2021
↑ aae-miR-927	DENV-2 (New Guinea C strain)	C6/36	↓ FLN	Promote virus replication	Avila-Bonilla et al., 2020
↓ miR-155	DENV-2 (PL046 strain)	Huh-7	↑ BACH1	Promote virus replication	Su et al., 2020
↑ miR-148a	DENV-2 (New Guinea C strain), DENV-NS1	CHME3	↓ USP33	Promote virus replication Promote inflammation	Mishra et al., 2020
↑ aae-miR-375	DENV-2 (New Guinea C strain)	Aag2	↑ CACTUS ↓ REL1	Promote virus replication	Hussain et al., 2013
↑ aae-miR-4728-5p	DENV-2 (New Guinea C strain)	C6/36	—	Promote virus replication	Su et al., 2017
↑ mir-150	DENV-2 (New Guinea C strain)	PBMCs from DHF patients	↓ SOCS1	Biomarker	Chen et al., 2014
↑ let-7c	DENV-2 (New Guinea C strain), DENV4 (V3361–1956 strain)	Huh7, U937-DC-SIGN	↓ BACH1	Inhibit virus replication	Escalera-Cueto et al., 2015
↑ miR-30e*	DENV-1 (Hawaii strain), DENV-2 ( New Guinea C strain), DENV-3 (H241 strain)	HeLa, U937, PBMCs	↓ IκBα	Inhibit virus replication	Jiang et al., 2012; Zhu et al., 2014
miR-34a	DENV-2 (New Guinea C strain)	HeLa	↓ Wnt/β-catenin pathway	Inhibit virus replication	Smith et al., 2017
↑ miR-3614-5p	DENV-2 (16681 strain)	Primary human macrophages	↓ ADAR1	Inhibit virus replication	Diosa-Toro et al., 2017
↓ miR-378	DENV-infected patients	Natural killer cells	↑ GrzB	Inhibit virus replication	Liu et al., 2016
↑ miR-424	DENV-2 (New Guinea C strain)	HeLa	↓ SIAH1	Inhibit virus replication	Smith et al., 2017; Murphy Schafer et al., 2020

*^a^ – The specific target of this miRNA has not been reported.*

*miRNAs are derived from human/mouse cells, unless otherwise noted.*

*↑ miRNA/gene expression is increased after a particular virus infection.*

*↓ miRNA/gene expression is decreased after a particular virus infection.*

*STMN1, Stathmin 1; TRAF6, TNFR-associated factor 6; FLN, Filamin; BACH1, BTB domain and CNC homolog 1; SOCS1, Suppressor of cytokine signaling 1; USP33, Ubiquitin-specific peptidase 33; IκBα, I kappa B alpha; ADAR1, Adenosine deaminase acting on RNA 1; GrzB, Granzyme B; SIAH1, Siah E3 ubiquitin protein ligase 1.*

Non-coding regions of flavivirus, the 5′UTR and 3′UTR, have significant impact in translation, replication and assembly of virus. miR-484, miR-744, and miR-133a are downregulated at early stage during DENV infection in Vero cells (Castillo et al., 2016; Castrillón-Betancur and Urcuqui-Inchima, 2017). Moreover, these three miRNAs facilitate DENV replication through targeting and increasing the 3′UTR of viral genome. Furthermore, miR-281 and miR-548g-3p have been shown to complement the 5′UTR of DENV, but when both two miRNAs are up-regulated, their effects on viral proliferation are opposite (Pasquinelli, 2012; Zhou et al., 2014; Wen et al., 2015). miR-281, which is abundantly expressed in the female mosquitoes’ midgut, promotes the replication of DENV by increasing its 5′UTR, while the up-regulated miR-548g-3p inhibits viral genome amplification by impairing DENV 5′UTR. It is worth mentioning that the mechanism of miR-281 is contrast to the classical pathways in which miRNAs interact with target genes to degrade mRNA or inhibit translation. miRNAs can also bind complementary to coding regions of viral genomes, although this is less common. Down-regulation of miR-252 by a synthetic inhibitor enhances DENV-2 transmembrane envelop protein (E protein), a protein involved in virus invasion, as well as virus titers (Yan et al., 2014). miR-21 shows a trend of up-regulation after DENV infection, and promotes DENV-2 proliferation in HepG2 cells. Unfortunately, the mechanism remains unclear, but may be related to the potential interaction of miR-21 with viral NS1 sequence (Miranda et al., 2006; Kanokudom et al., 2017). NS1 exerts multiple roles in the viral life cycle, especially in virus propagation and immune evasion of the complement pathway (Glasner et al., 2018).

More recently, it has been achieved to control the virus tissue tropism by incorporating a specific miRNA recognition element (MRE) from the viral genome into engineered viruses. This has been implemented as a neoteric strategy to exploit secure vaccines against pathogenic viruses (Wong et al., 2020). For instance, inserting the MRE for the hepatic-specific miR-122 in the 3′UTR of DENV replicon could block DENV infection of vital tissues/organs (Lee et al., 2010). Similarly, the insertion of the MRE for the hematopoietic specific miR-142 into the DENV-2 genome restricts replication of the virus in dendritic cells and macrophages, but not in non-hematopoietic cell types (Pham et al., 2012).

### miRNAs Modulate Host Factors to Influence Dengue Virus Replication

After DENV infection, miRNAs can regulate series of key genes related to innate immunity. For example, miR-146a-targeted TNFR-associated factor 6 (TRAF6) and miR-155-targeted BTB domain and CNC homolog 1 (BACH1) can promote viral replication by dampening IFN-β (Wu et al., 2013; Arboleda et al., 2019; Su et al., 2020). TRAF6, which mediates multiple immune signaling pathways, is essential for the production of type I IFN, and the characteristic of miR-146a is consistent with reports in JEV and ZIKV (Wu et al., 2013; Sharma et al., 2015; Shukla et al., 2021; Xie et al., 2021). Furthermore, miR-146a is also associated with anti-inflammatory effects in JEV and ZIKV infection, but has not been reported in DENV. Pro-DENV activity of downregulated miR-155 is due to augment BACH1, and thus contributing to the production of the heme oxygenase-1 (HO-1) mediated promotion of DENV NS2B/NS3 protease activity and inhibit IFN responses (Pulkkinen et al., 2011; Su et al., 2020). Nevertheless, let-7c exerts a role in the restriction of viral replication by down-regulating BACH1 (Escalera-Cueto et al., 2015). The depletion of miR-223 or overexpression of Stathmin 1 (STMN1) enhances DENV2 replication in EAhy926 cells (a human endothelial-like cell line) (Wu et al., 2014). Function as a microtubule (MT)-destabilizing protein, up-regulated STMN1 is likely to influence actin function by changing MT dynamics, which provides advantageous conditions for virus invasion. Abundant extracellular vesicles (EVs) load with miR-148a in DENV-infected monocytes and DENV-NS1-transfected cells. Whereafter, miR-148a represses the expression levels of Ubiquitin-specific peptidase (USP33), which in turn weakens the stability of ATF3 protein, an inhibitor molecule of pro-inflammatory pathways, by deubiquitylation, ultimately preparing the uninfected cells for more favorable DENV infection and triggering a massive production of proinflammatory genes (Mishra et al., 2020). miR-375, miR-927, and miR-4728-5p are detected and their expression levels increased in the blood fed mosquitoes infected DENV (Hussain et al., 2013; Su et al., 2017; Avila-Bonilla et al., 2020). miR-375 controls two genes involved in immunity, CACTUS versus REL1 via targeting their respective 5′UTR but not 3′UTR and further suppresses phosphorylation of the NF-kB, it is possible to enhance DENV infection. Similarly, the virus utilizes miR-927 and miR-4728-5p to boost its own proliferation, but neither miR-927 nor miR-4728-5p are known about the specific mechanism of DENV-host interaction.

Previous studies have clarified overexpressed miR-30* and miR-424 knockdown IκBα and Siah E3 ubiquitin protein ligase 1 (SIAH1) genes related to inherent immunity. Subsequently, miR-30* mediated NF-κB/IκBα negative feedback loop is disrupted with an excessive activation of NF-κB and increases IFN-β and the downstream ISGs, eventually leading to inhibition of DENV replication (Jiang et al., 2012; Zhu et al., 2014). As an E3 ubiquitin ligase, SIAH1 generally induces DENV infection by activating the unfolded protein response (UPR). While SIAH1 expression is suppressed, proliferation of DENV is also restricted (Murphy Schafer et al., 2020). DENV replication is reduced by miR-3614-5p overexpression, a miRNA that binds to and degrades the DENV pro-viral protein Adenosine Deaminase Acting on RNA 1 (ADAR1) in macrophages (Diosa-Toro et al., 2017), while down-regulated miR-378 achieves the same effect in DENV-infected NK cells by promoting granzyme B (GrzB), a serine protease released by NK cells that inhibits viruses (Liu et al., 2016). miR-34 family, as valid anti-flaviviral miRNAs, has been confirmed that miR-34a mimic transfection effectively impaired infection by DENV, JEV and WNV(Smith et al., 2017). Another member of the miR-34 family, miR-34c, also plays a role in neurogenesis and pro-inflammatory response, which will be elucidated in JEV, WNV and ZIKV in detail.

## Regulation of miRNAs in Japanese Encephalitis Virus Infection

Japanese encephalitis virus is a neurotropic virus that infects the Central Nervous System (CNS) and causes fatal encephalitis. Accumulating evidence points to microglia activation and bystander damage as the main pathogenesis of Japanese encephalitis and its complications (Chang et al., 2021). After viral infection, miRNAs expression alters mostly occur in human/mouse microglia and subsequently respond to viral invasion by influencing viral replication and inflammation in the body.

### miRNAs Modulate Host Factors to Influence Japanese Encephalitis Virus Replication

miR-301a (Hazra et al., 2017), miR-22 (Zhou et al., 2020), miR-432 (Sharma et al., 2016), miR-370-5p (Li W. et al., 2016), miR-374b-5p (Rastogi and Singh, 2019) are shown to exert the proviral effect by directly targeting immunity-related genes or interferon-induced genes, such as IRF1, SOCS5, MAVS, STAT1, and PTEN, which further mediated the expression of type I IFN (Table 2). It is quite interesting that down-regulated miR-370-5p and up-regulated miR-374b-5p increase the level of type I interferon in JEV infected cells, while other miRNAs above mentioned promote JEV replication by impairing the level of it. Moreover, miR-33a-5p degradation contributes to viral replication via increasing the intracellular EEF1A1, an interaction partner of the JEV proteins NS3 and NS5 in replicase complex (Chen et al., 2016). Jiang et al. (2020) also found JEV NS3 can suppress the expression level of miR-466d-3p and enhance JEV amplification by targeting IL-1β, but the mechanism remains to be explored.

**TABLE 2:** Differentially expressed miRNAs involved in JEV infection and their targets*^a^*.

microRNA	Virus-types	Host system	Target	Effect	References
↓ miR-370-5p	JEV(SA14-14-2 strain)	U251	—	Promote virus replication	Li W. et al., 2016
↑ miR-374b-5p	JEV (JaOArS982 strain)	Human microglial cells	↓ PTEN	Promote virus replication	Rastogi and Singh, 2019
↓ miR-466d-3p	JEV(P3 strain)	BV2, NA	↑ IL-1β	Promote virus replication	Jiang et al., 2020
↓ miR-432	JEV (JaOArS982 strain)	CHME3	↑ SOCS5	Promote virus replication	Sharma et al., 2016
↑ miR-146a	JEV (JaOArS982 strain)	CHME3	↓ STAT1, TRAF6, IRAK1, IRAK2	Promote virus replication Anti-inflammatory	Sharma et al., 2015
↓ miR-33a-5p	JEV(P3 strain)	HEK293T	↑ EEF1A1	Promote virus replication	Chen et al., 2016
↑ miR-22	JEV NS1’ defective viruses (rG66A strain)	HeLa	↓ MAVS	Promote virus replication	Zhou et al., 2020
↑ miR-301a	JEV(GP78 strain)	HT22 CHME3, BV2	↓ SOCS5, IRF1 ↓ NKRF	Promote virus replication Promote inflammation	Hazra et al., 2017, 2019
↑ miR-125b-5p	JEV(RP-9 strain)	BHK-21	↓ STAT3, MAP2K7, TRIAP1	Inhibit virus replication	Huang et al., 2021
↑ ssc-miR-124	JEV(SA14-14-2 strain)	PK15	↓ DNM2	Inhibit virus replication	Yang et al., 2016
miR-34a	JEV (SA-14-2-8 strain)	HeLa	↓ Wnt/β-catenin pathway	Inhibit virus replication	Smith et al., 2017
↑ miR-155	JEV(P20778 strain) JEV(GP78 strain)	CHME3 BV-2, Mice brain tissues	↓ IRF8, ↑ CD45 ↓ SHIP1	Inhibit virus replication Promote inflammation	Pareek et al., 2014; Thounaojam et al., 2014b
↑ miR-29b	JEV(GP78 strain)	BV2	↓ TNFAIP3	Promote virus replication Promote inflammation	Thounaojam et al., 2014a
↓ miR-326-3p	JEV(P3 strain)	BV2	↑ BCL3, MK2, TRIM25	Promote inflammation	Li et al., 2020
↑ miR-19b-3p	JEV(P3 strain)	U251, BV2, Mice brain tissues	↓ RNF11	Promote inflammation	Ashraf et al., 2016
↑ miR-15b	JEV(P3 strain)	U251, BV2, Mice brain tissues	↓ RNF125	Promote inflammation	Zhu et al., 2015
↑ let-7a/b	JEV(GP78 strain)	N9, N2A, Mice brain tissues	↑ NOTCH-TLR7 pathway	Promote inflammation	Mukherjee et al., 2019
↓ miR-34c-5p	JEV (JaOArS982 strain, P20778 strain)	CHME3	↑ NOTCH1	Promote inflammation	Kumari et al., 2016

*^a^ – The specific target of this miRNA has not been reported. miRNAs are derived from human/mouse cells, unless otherwise noted.*

*↑ miRNA/gene expression is increased after a particular virus infection.*

*↓ miRNA/gene expression is decreased after a particular virus infection.*

*PTEN, phosphatase and tensin homolog; IL-1β, interleukin 1β; SOCS5, Suppressor of cytokine signaling 5; STAT1/3, Signal transducer and activator of transcription 1/3; TRAF6, TNF receptor-associated factor 6; IRAK1/2, IL-1 receptor associated kinase-1/2; EEF1A1, Eukaryotic translation elongation factor 1A1; MAVS, Mitochondria antiviral signaling protein; IRF1/8, Interferon regulatory factor 1/8; NKRF, NF-κB repressing factor; MAP2K7, mitogen-activated protein kinase 7; TRIAP1, TP53 regulated inhibitor of apoptosis 1; DNM2, dynamin 2; SHIP1, Src homology 2-containing inositol phosphatase 1; TNFAIP3, Tumor necrosis factor alpha-induced protein 3; BCL3, B-cell CLL/lymphoma 3; MK2, MAPK-activated protein kinase 2; TRIM25, Tripartite motif-containing protein 25; RNF11/125, Ring finger protein 11/125; TLR7, Toll Like Receptor 7.*

Persistently JEV infection increases miR-125b-5p expression and limits viral replication by dampening STAT3, MAP2K7, TRIAP1 production, which genes are associated with apoptosis and cell growth (Huang et al., 2021). Overexpression of miR-124 in PK-15 cells is found to decrease the expression level of DNM2, a GTPase responsible for vesicle scission, further impairing JEV proliferation (Yang et al., 2016). Work by Pareek et al. (2014) delineated that increased miR-155 levels versus decreased IRF8 levels can dramatically inhibit NF-κB signaling pathway and JEV multiplication, while the effect of miR-146a on virus inhibition is not significant. On the contrary, reports by Sharma et al. (2015) have shown that miR-146a participates in the reduction of NF-κB activity by inhibiting specific genes (STAT1, IRAK1, IRAK2, TRAF6) in the TLR mediated NF-κB pathway. Subsequently this miRNA exerts an anti-inflammatory role and promote viral replication, similar cases appear in ZIKV infection as well (Shukla et al., 2021).

### miRNAs Play a Pro-inflammatory Role During Japanese Encephalitis Virus Infection

During JEV infection, host miRNAs can not only regulate JEV replication, but also increase microglial activation and accelerate the production of inflammatory cytokines by influencing important genes in the pro-inflammatory pathway (Lawrence, 2009; Thounaojam et al., 2014a,b; Ashraf et al., 2016; Hazra et al., 2019; Mukherjee et al., 2019). Highly expressed miR-15b (Zhu et al., 2015) and miR-155 (Thounaojam et al., 2014b) are considered to exert the pro-inflammatory effect by directly targeting the 3′UTR of RNF125 and SHIP1 respectively, leading to the degradation of both two genes. This results in the subsequent phosphorylation of NF-κB (Lawrence, 2009). Similarly, significantly increased expression level of miR-19b-3p (Ashraf et al., 2016), miR-29b (Thounaojam et al., 2014a), and miR-301a (Hazra et al., 2019) stimulate microglia secrete inflammatory mediators in JEV-infected BV2 cells, including TNF-α, IL-6, and IL-1β, via separately decreasing RNF11, TNFAIP3 and NKRF, which are all negative regulators of NF-κB signaling pathway. Mukherjee et al. (2019) substantiated that JEV infection results in a time-dependent increase in the let-7a/b expression, both two miRNAs act as a TLR7 ligand and activate NOTCH, a pathway involved inflammatory cytokine production. Simultaneously, they also found down-regulation of miR-34b-5p increases NOTCH1 expression challenged with JEV, which is contrary to the upregulation of this miRNA expression in WNV infection (Kumari et al., 2016).

## Regulation of miRNAs in West Nile Virus Infection

West Nile virus belongs to the JEV serotype, which maintains in the environment by an avian–mosquito life cycle (Barrows et al., 2018). The neuro-invasive disease of WNV can be fatal, or recovery from the infection may require specialized care, with symptoms lasting more than a year (Petersen et al., 2013). Such neurological symptoms are also reflected in the concentration of targets of differentially expressed miRNAs.

### miRNAs Modulate Host Factors to Influence West Nile Virus Replication

Slonchak et al. (2014, 2015) manifested that up-regulated miR-532-5p and down-regulated miR-2940 suppress WNV proliferation in cells by inhibiting their target genes SESTD1, TAB3 and MetP, all of which are required for efficient WNV replication (Table 3). Hs_154, is significantly produced by WNV in various cell lines and occurred *in vivo*. Upregulation of Hs_154 and subsequent suppression of CTCF and ECOP, two genes related to anti-apoptosis, cause a significant reduction in viral replication through facilitating WNV-dependent induction of apoptosis (Smith et al., 2012).

**TABLE 3:** Differentially expressed miRNAs involved in WNV infection and their targets^a^.

microRNA	Virus-types	Host system	Target	Effect	References
↑ miR-532-5p	WNV_KUN_ (NSW2011 strain)	HEK293, Mice brain tissues	↓ SESTD1, TAB3	Inhibit virus replication	Slonchak et al., 2015
↓ aae-miR-2940-5p	WNV_KUN_	C6/36	↓ MetP	Inhibit virus replication	Slonchak et al., 2014
↑ Hs_154	WNV(385-99 strain)	HEK293, SK-N-MC	↓ CTCF, ECOP	Inhibit virus replication	Smith et al., 2012
miR-34a	WNV(385-99 strain)	HeLa	↓ Wnt/β-catenin pathway	Inhibit virus replication	Smith et al., 2017
↑ miR-155	WNV(NY99 strain, EG101 strain)	SK-N-SH, Mice brain tissues	↓ IL-13, BDNF ↑ CCR9(predictive)	Inhibit virus replication Promote inflammation	Kumar and Nerurkar, 2014; Natekar et al., 2019
↑ miR-32	WNV(NY99 strain)	Mice brain tissues	↓ SMAD6, SOX4, IL36B, ↑ CCR9(predictive)	Neuroinflammatory	Kumar and Nerurkar, 2014
↓ miR-449c	WNV(NY99 strain)	Mice brain tissues	↑ CXCL10, CXCL11, NFKBIA, SERPINE1, IL2RB, CCR1, MYC, SNAI1, BCL6 ↓ EPHB2 (predictive)	Neuroinflammatory	Kumar and Nerurkar, 2014
↓ miR-196a	WNV(NY99 strain)	Mice brain tissues	↑ CCR2, NFKBIA ↓ SMAD6 (predictive)	Neuroinflammatory	Kumar and Nerurkar, 2014
↓ miR-202-3p	WNV(NY99 strain)	Mice brain tissues	↑ TNFRSF1B, CCR7, BCL2L1, S100A8, THBS1, CCL7, IL10 ↓ IL13 (predictive)	Neuroinflammatory	Kumar and Nerurkar, 2014
↓ miR-125a-3p	WNV(NY99 strain)	Mice brain tissues	↑ PTGS2, IL1R1, IL10, CCL4 (predictive)	Neuroinflammatory	Kumar and Nerurkar, 2014
↑ aae-miR-92	WNV(NY99 strain)	C7/10	—	Embryogenesis	Skalsky et al., 2010
↓ aae-miR-989	WNV(NY99 strain)	C7/10	—	Female-specific miRNA	Skalsky et al., 2010)
↑ miR-34c -5p	WNV(NY99 strain)	MEFs	—	—	Kumari et al., 2016

*^a^ – The specific target of this miRNA has not been reported.*

*miRNAs are derived from human/mouse cells, unless otherwise noted.*

*↑ miRNA/gene expression is increased after a particular virus infection.*

*↓ miRNA/gene expression is decreased after a particular virus infection.*

*GATA4, GATA binding protein 4; SESTD1, SEC14 and spectrin domain containing 1; TAB3, TGF-beta activated kinase 1 binding protein 3; MetP, Metalloprotease m41 ftsh; CTCF, CCCTC-binding factor; ECOP, Epidermal growth factor receptor (EGFR)-coamplified and overexpressed protein; IL-1R1/2RB/10/13/36B, Interleukin 1R1/2RB/10/13/36B; BDNF, Brain-derived neurotrophic factor; CCR1/2//7/9, CC motif chemokine receptor 1/2//7/9; CXCL10/11, C-X-C motif chemokine ligand 10/11; CCL4/7, CC motif chemokine ligand 4/7; SMAD6, SMAD family member 6; SOX4, SRY-related HMG box 4; NFKBIA, Nuclear factor-kappa-B inhibitor-alpha; SERPINE1, Serine protease inhibitor clade E member 1; SNAI1, Snail family zinc finger 1; EPHB2, Erythropoietin-producing hepatoma-amplified sequence receptor transmembrane tyrosine kinase; BCL2L1/6, B-cell CLL/lymphoma 2L1/6; MRP8, Myeloid-related protein 8; THBS1, Thrombospondin-1; PTGS2, Prostaglandin endoperoxidase synthase 2; COX-2, Cyclooxygenase-2; TNFRSF1B, TNF receptor superfamily member 1B gene; TNFR2, Tumor necrosis factor receptor 2.*

### miRNAs Are Associated With Neuroinflammation Caused by West Nile Virus Infection

Kumar and Nerurkar (2014) conducted an integrated analysis of miRNAs and their disease related targets in mice brain with WNV infection. The network of miRNA interactions with presumptive target genes is demonstrated that miR-196a, miR-202-3p, miR-449c, and miR-125a-3p are downregulated, while miR-155 and miR-32 are adverse. All these miRNAs are positive correlated with protein levels of neuroinflammatory genes. In addition, WT and miR-155^–/–^ mice was selected to further and affirm the role of miR-155 in suppressing virus replication and promoting anti-viral immune response, which is similar to JEV, but the target gene for its action has yet to be identified.

## Regulation of miRNAs in Zika Virus Infection

Zika virus was first isolated from the blood of a non-human primate in 1947 and from *Aedes africanus mosquitoes* 1 year later, which is mainly transmitted by *Aedes albopictus* and *Aedes aegypti* (Musso and Gubler, 2016; Lin et al., 2018). As shown in a variety of experimental models and human samples, ZIKV infection induces neuronal apoptosis and disrupts cellular homeostasis, resulting in microcephaly, anencephaly, ventricular enlargement and calcification (Polonio and Peron, 2021).

### miRNAs Modulate Host Factors to Influence Zika Virus Replication

miR-142-5p has been confirmed to be a factor that accelerates the expression of IL-6 signal transducer (L6ST) and Integrin subunit alpha V (ITGAV), then inhibits ZIKV replication, possibly through activation of innate immune antiviral response and phagosome pathway (Seong et al., 2020; Table 4).

**TABLE 4:** Differentially expressed miRNAs involved in ZIKV infection and their targets^a^.

MicroRNA	Virus-types	Host system	Target	Effect	References
↑ miR-146a	ZIKV-NS1 (#ZIKVSU-NS1)	HMC3	↓ TRAF6, STAT1	Promote virus replication Anti-inflammatory	Shukla et al., 2021
↓ miR-142-5p	ZIKV (MR766 strain, PRVABC59 strain)	hUCMSC	↑ IL6ST, ITGAV	Inhibit virus replication	Seong et al., 2020
miR-34a	ZIKV (PRVABC59 strain)	HeLa	↓ Wnt/β-catenin pathway	Inhibit virus replication	Smith et al., 2017
miR-627–5	ZIKV (MR766 strain)	Human fetal brains	Viral NS5, MCPH1, MCPH3, MCPH5 (predictive)	Neurogenesis, Microcephaly	McLean et al., 2017
miR-4646	ZIKV (MR766 strain)	Human fetal brains	Viral NS1, MCPH2 (predictive)	Neurogenesis, Microcephaly	McLean et al., 2017
miR-1304	ZIKV (MR766 strain)	Human fetal brains	Viral E, MCPH3, MCPH8, MCPH9, MCPH10, MCPH12 (predictive)	Neurogenesis, Microcephaly	McLean et al., 2017
miR-6771	ZIKV (MR766 strain)	Human fetal brains	Viral E, MCPH6 (predictive)	Neurogenesis, Microcephaly	McLean et al., 2017
miR-4528	ZIKV (MR766 strain)	Human fetal brains	Viral NS5, MCPH7 (predictive)	Neurogenesis, Microcephaly	McLean et al., 2017
miR-3198	ZIKV (MR766 strain)	Human fetal brains	Viral E, MCPH11 (predictive)	Neurogenesis, Microcephaly	McLean et al., 2017
↑ miR-1273g-3p	ZIKV E	HeLa, fNSCs	↓ NOTCH2	Neurogenesis	Bhagat et al., 2018
↑ miR-204-3p	ZIKV E	HeLa, fNSCs	↓ PAX3	Neurogenesis	Bhagat et al., 2018
↑ miR-204-5p	ZIKV E	fNSCs	↓ WNT2	Neurogenesis	Bhagat et al., 2021
↑ miR-34c	ZIKV(H/PF/2013 strain)	GSCs	↓ BCL2, NOTCH, NUMB	Neurogenesis	Iannolo et al., 2019
↑ miR-145	ZIKV (Brazilian PE strain)	SH-SY5Y, Stillborn brains	↓ ACTG1, ACTB, CDK6, DAG1, LRP2, TCF4 (predictive)	Microcephaly	Castro et al., 2019
↑ miR-148a	ZIKV (Brazilian PE strain)	SH-SY5Y, Stillborn brains	↓ ALCAM, LDB1, LOX, ERBB4, EFNA3, NRP1, ROBO2, XRN1, SIX4, ZFHX4 (predictive)	Microcephaly	Castro et al., 2019
↑ miR-124-3p	ZIKV(MR766 strain, Paraiba strain)	hNSCs	↓ TFRC	Microcephaly	Dang et al., 2019
↑ miR-9	ZIKV(SZ01 strain)	Mice brain tissues	↓ GDNF	Microcephaly	Zhang et al., 2019
↑ miR-101-3p	ZIKV-NS1 (#ZIKVSU-NS1)	hBMVEC, hCMEC/D3	↓ VE-cadherin	Damage the barrier integrity of human brain microvascular endothelial cells	Bhardwaj and Singh, 2021
↑ miR-7013-5p	ZIKV(HS-2015-BA-01 strain)	N2A	↓ Nr4a3	Neuronal dysfunction	Alpuche-Lazcano et al., 2021

*^a^miRNAs are derived from human/mouse cells, unless otherwise noted.*

*↑ miRNA/gene expression is increased after a particular virus infection.*

*↓ miRNA/gene expression is decreased after a particular virus infection.*

*TRAF6, TNF receptor-associated factor 6; STAT1, Signal transducer and activator of transcription 1; IL6ST, Interleukin-6 signal transducer; ITGAV, Integrin subunit alpha V; MCPH 1-12, Microcephalin 1-12; PAX3, Paired box 3; BCL2, B-cell CLL/lymphoma 2; ACTG1, Actin Gamma 1; ACTB, Actin Beta; CDK6, Cyclin D-dependent kinase 6; DAG1, Dystrophin-associated glycoprotein 1; LRP2, LDL Receptor Related Protein 2; TCF4, Transcription Factor 4; ALCAM, Activated leukocyte cell adhesion molecule; LDB1, LIM Domain Binding 1; ERBB4, Erb-b2 receptor tyrosine kinase 4; HER4, Heregulin 4; EFNA3, Ephrin A3; NRP1, Neuropilin 1; XRN1, 5′-3′ Exoribonuclease 1; SIX4, Sine oculis homeobox 4; ZFHX4, Zinc Finger Homeobox 4; TFRC, Transferrin receptor gene; GDNF, Glial cell-derived neurotrophic factor; VE-cadherin, Vascular endothelial cadherin; Nr4a3, Three members of the orphan nuclear receptor 4.*

Moreover, due to the close relationship between ZIKV and mosquitoes, multiple studies reported changes in miRNA expression in mosquitoes after ZIKV infection. Saldaña et al. (2017) and Yen et al. (2019) predicted the putative binding sites of three arboviruses (CHIKV, DENV, ZIKV) to mosquito-derived miRNAs, and found eight miRNA-binding sites have low minimum free energy (MFE), suggesting they are more likely to constitute miRNA-viral RNA complexes and subsequently participate in viral replication theoretically.

### miRNAs Are Associated With Neurogenesis Caused by Zika Virus Infection

Based on ZIKV’s special preference for neural stem cells (NSCs), this cell is the best choice to study the effects of ZIKV infection on neurogenesis *in vitro*. Bhagat et al. revealed miR-1273g-3p as well as miR-204-3p are upregulated in response to ZIKV envelope E protein, a viral component responsible for attachment and entry into host cell (Bhagat et al., 2018). Further, these two miRNAs directly influenced developmental genes PAX3 and NOTCH2 respectively, which contributes to developmental defects in fNSCs (Bhagat et al., 2018). Interestingly, miR-204-5p, also a member of the miR-204 family, constitutes the miR-204-5p/WNT2 axis after ZIKV E protein invasion, and is involved in ZIKV induced impairment in the proliferation and immature differentiation of NSCs. miR-34c was identified as a ZIKV-regulated miRNA with a potential role in restricting NSC growth (Iannolo et al., 2019). This may be dependent on the inhibition of in anti-apoptosis gene Bcl2 and genes involved in stemness maintenance and nervous system development, such as NOTCH and NUMB.

Zika virus infection was found to increase the incidence of microcephaly in fetuses born to mothers infected with ZIKV in 2015. Then the association of ZIKV infection and microcephaly was confirmed in a ZIKV-infected mouse model the following year (Cugola et al., 2016; Li C. et al., 2016; Li H. et al., 2016; Mlakar et al., 2016; Shao et al., 2016). Current studies have shown that miRNA-mRNA network is dysregulated during ZIKV infection, resulting in microcephaly. By evaluating neurosphere growth dynamics, it is speculated that miR-124-3p mediates microcephaly by inhibiting its target gene related to cell-cycle regulation TFRC (Dang et al., 2019). Zhang et al. (2019) demonstrated miR-9 is overexpressed after ZIKV infection and identified down-regulated Glial cell-derived neurotrophic factor (GDNF) is a target of miR-9, GDNF can protect neural progenitors from miR-9-induced apoptosis, which perhaps related to the microcephaly phenotype. Two upregulated miRNAs, miR-145 and miR-148a, are screened involving in cellular migration in brain samples from stillborn with congenital Zika syndrome and 13 common targets genes related to microcephaly, lissencephaly and other neuronal malformation for both miRNAs are predicted by bioinformatics software (Castro et al., 2019). Another bioinformatics study predicted potential genome-wide binding sites between miRNA and ZIKV, and found significant homology between 6 miRNAs and ZIKV genome and 12 autosomal recessive primary microcephaly (MCPH) genes, which may contribute to MCPH production during human fetal brain development (McLean et al., 2017).

## Regulation of miRNAs in Other Flaviviruses Infection

In addition to the above widely influential zoonoses, other members of the *Flavivirus* genus whose infection is also closely related to cellular miRNA. TMUV is an emerging and re-emerging zoonotic pathogen that adversely affects poultry industry in recent years, especially the duck breeding industry (Yang et al., 2021). Cui et al. (2018, 2020) and Huang et al. (2020) first employed deep sequencing to investigate miRNA profiles in DEF cells after DTMUV infection and revealed a novel host evasion mechanism adopted by DTMUV via miR-221-3p inhibiting the negative regulator of host immune response, SOCS5. Subsequently, this miRNA hinders JAK-STAT signaling mediated IFN response and promote virus replication, while miR-148a-5p has a similar effect on the virus replication by modulating SOCS1. TBEV causes neurological symptoms that can lead to permanent disability or death. Although there have been no studies on miRNA expression after TBEV infection, several studies have shown that host-specific miRNAs target sequences integrated into viral genomes (such as tick-specific miR-275 and neuro-specific miR-124a) can effectively control the escape of TBEV chimeric viruses or LGTV (a naturally attenuated member of the TBEV serum complex) and reversal of neurotoxic phenotype (Heiss et al., 2011, 2012; Tsetsarkin et al., 2016). Hermance et al. (2019) confirmed that miRNA changes can regulate Powassan virus (POWV) replication in the host through high-throughput sequencing and transfection of specific miRNA inhibitors *in vitro*.

## Other Non-Coding RNAs During Flavivirus Infection

Since lncRNA/circRNA can competitively bind with miRNA, the expression level of target mRNA can be indirectly regulated through miRNA. ERG-associated lncRNA (lncRNA-ERGAL) was proved to be involved in promoting stability and integrity of vascular endothelial barrier during DENV infection by sponging miR-183-5p (Zheng et al., 2020). Li et al. (2020) established circRNA–miRNA–mRNA interaction network following JEV infection and unraveled cirC_0000220 binds to miR-326-3p to restrict BCL3, MK2 and TRIM25 mediated inflammatory response.

sfRNA is a product of incomplete degradation of viral genomic RNA by host cellular 5′-3′ exonuclease XRN-1 (Slonchak and Khromykh, 2018). However, the production of sfRNA is limited to *Flavivirus* genus and not found in the other two genera (Schnettler et al., 2012). This non-coding RNA generally promotes flavivirus pathogenesis by impairing the IFN response, as well documented in DENV, WNV (Schuessler et al., 2012; Pompon et al., 2017). But the specific affinity between ZIKV sfRNA and mosquito DEAD-Box helicase ME31B inhibits viral RNA replication and virion production (Göertz et al., 2019). Only when loaded into Argonaute (AGO), siRNAs and miRNAs perform their functions. Hence, Zeng et al. (2021) constructed AGO-associated viral siRNA profiles and completed a network between ZIKV siRNAs and their probable viral RNA (vRNA) targets via AGO-associated RNA sequencing. Twenty-nine siRNAs and 114 potential targets were obtained from the ZIKV genome, and further functional characterization of ZIKV siRNAs need to be verified. Hussain and Asgari (2014) obtained six miRNA-like vsRNAs mapped to the viral 5′ and 3′UTR regions, termed vsRNA1-6, in *Ae. aegypti* cells after DENV-2 infection. As vsRNA5 has the most significant effect on virus replication, it was selected for further study. The results showed that vsRNA5 with characteristics similar to miRNAs with AGO-2 targets the viral genome NS1 and inhibits viral replication. The same research group also utilized bioinformatics analysis to identify and coin a WNV-encoded small regulatory viral RNA, KUN-miR-1, from viral 3′UTR (Hussain et al., 2012). The transcription factor GATA4 is identified as a target of KUN-miR-1. GATA mRNA expression also increases after KUN-miR-1 RNA mimic is transfected into *Ae. aegypti* C6/36 cells, which seems to be necessary for efficient WNV replication.

## Concluding Remarks

To understand host–virus interactions is critical to antiviral response. Based on miRNAs regulate a variety of intracellular biological pathways, including virus infection, which has been demonstrated in many viruses (Patil and Karpe, 2020; Song et al., 2020; Zhao et al., 2021). Hence, we emphasized the interaction between flavivirus and host miRNA. Two primary regulatory approaches are adopted by miRNAs in response to viral infection. Due to the high mutation rate of RNA viruses, antiviral effects resulting from direct binding of miRNAs to viral genomes may be rare, unless the viral genome is extremely capable of binding to miRNA (Trobaugh and Klimstra, 2017). This may well explain the lack of coverage, only in DENV (Zhou et al., 2014; Wen et al., 2015; Castillo et al., 2016; Castrillón-Betancur and Urcuqui-Inchima, 2017). Moreover, it has been widely reported that viruses can manipulate the expression levels of specific miRNAs to establish an environment conducive to virus survival and transmission (Hussain et al., 2013; Wu et al., 2014; Chen et al., 2016; Sharma et al., 2016). Meanwhile, the host can also interrupt the virus life cycle by altering miRNA expression levels. In addition to host miRNA, host/virus also produces some other non-coding RNAs during viral infection, which all play different roles in response to viral invasion (Hussain et al., 2012; Ding et al., 2018; Göertz et al., 2019; Zheng et al., 2020; Zeng et al., 2021).

In this view, we collected the differentially expressed miRNAs infected by *Flavivirus* genus members, mainly DENV, JEV, WNV, and ZIKV. Through the classification of regulatory effects, it is found that these miRNAs principally participate in the process of viral replication, neuroinflammation, neurogenesis and so on (Escalera-Cueto et al., 2015; Liu et al., 2016; Castrillón-Betancur and Urcuqui-Inchima, 2017; Dang et al., 2019; Hazra et al., 2019; Mukherjee et al., 2019; Bhagat et al., 2021). Furthermore, several miRNAs show identical or opposite expression by different flavivirus infection and thus play similar or opposite roles. miR-146a, upregulated after DENV, JEV and ZIKV infections, promotes viral replication by targeting TRAF6 in different cell lines (Wu et al., 2013; Sharma et al., 2015; Shukla et al., 2021). As a member of the antiviral miR-34 family, miR-34c is down-regulated after JEV infection, while up-regulated after WNV and ZIKV infection. But they all work by targeting the NOTCH gene, therefore, it can be speculated that one of the reasons for this differential expression is the difference in cell lines. Although the anti-viral effect of miR-155 has been extensively studied, its expression is reduced during DENV infection to promote viral survival, but with distinct outcomes in JEV and WNV (Pareek et al., 2014; Arboleda et al., 2019; Natekar et al., 2019; Su et al., 2020). Overall, miR-146a, miR-155, and miR-34 family are expected to be potential antiviral targets, the mechanism by which they control the virus should be further investigated in the future. Beyond that, expressing tissue-specific miRNA recognition element has been used as an effective mechanism to exploit attenuated vaccines, helping to reduce the transmission of flavivirus (Lee et al., 2010; Pham et al., 2012; Yen et al., 2018; Buchman et al., 2019). Based on the close association between flavivirus and vector mosquitoes, a large number of mosquito-derived miRNAs have been reported (Skalsky et al., 2010; Hussain et al., 2013; Avila-Bonilla et al., 2020). These host-specific miRNAs also rely on binding to host genes to regulate virus infection. It is worth mentioning that Zhu et al. (2021) discovered a human blood-derived miRNA (miR-150-5p), which interferes with mosquito antiviral system to promote flavivirus infection through cross-species RNAi mechanism, determining the transmission ability of flavivirus in nature.

Other host-derived RNAs, such as lncRNA and circRNA, often regulate target mRNAs by forming a functional grid together with miRNA. During flavivirus infection, lncRNA-ERGAL and cirC_0000220 can regulate the biological processes of infected cells by associating with miR-183-5p and miR-326-3p, respectively (Li et al., 2020; Zheng et al., 2020). But this type of reporting is still limited and a lot of work needs to be done. Increasing evidence points to that viruses themselves can produce non-coding RNAs in addition to their hosts, such as sfRNAs, siRNAs and viral miRNAs mentioned in this review. The interaction between these non-coding RNAs and host/virus has expanded the novel strategy to manage flavivirus (Hussain et al., 2012; Hussain and Asgari, 2014; Slonchak and Khromykh, 2018; Göertz et al., 2019).

In brief, we discussed the regulatory role of host miRNAs after infection with *Flavivirus* genus members, mainly through miRNA directly binds to the viral genome or vital host genes, although the latter is more widely reported. Understanding host–virus interactions will help us discover promising targets of antiviral drugs and viral therapeutic, which will provide new thinking directions for preventing viral infection.

## Author Contributions

WC and YP contributed ideas for the review, wrote the manuscript, and produced the figures. AC, MW, ZY, and RJ edited and revised the manuscript. All authors contributed to the article and approved the submitted version.

## Conflict of Interest

The authors declare that the research was conducted in the absence of any commercial or financial relationships that could be construed as a potential conflict of interest.

## Publisher’s Note

All claims expressed in this article are solely those of the authors and do not necessarily represent those of their affiliated organizations, or those of the publisher, the editors and the reviewers. Any product that may be evaluated in this article, or claim that may be made by its manufacturer, is not guaranteed or endorsed by the publisher.

## Abbreviations

5 ′ UTR: 5 ′ untranslated region
3 ′ UTR: 3 ′ untranslated region
DENV: Dengue virus
WNV: West Nile virus
ZIKV: Zika virus
JEV: Japanese encephalitis virus
YFV: Yellow Fever virus
TBEV: Tick-borne encephalitis virus
LGTV: Langat virus
TMUV: Tembusu
AGO: Argonaute
IFN: interferon
ISG: IFN-stimulating genes
TLRs: toll-like receptors
RIG-I: retinoic acid-inducible gene I
NF- κ B: nuclear factor- κ B
CHME3: human microglial cells
C6/36: *Ae. albopictus* cell line
C7/10: *Ae. Albopictus* cell line
THP-1: human monocytic cell line
hNSCs: human neural stem cells
fNSCs: fetal neural stem cells
GSCs: glioblastoma stem cells
N2A: neuro-2A
PK-15: porcine kidney-15 cells
MEFs: mouse embryonic fibroblasts
DEF: Duck embryo fibroblasts
PBMCs: peripheral blood mononuclear cells
HO-1: heme oxygenase-1.
